# Sex Differences in the Relationship Between Cortical Thickness and Sensory Motor Symptoms in Adults on the Autism Spectrum

**DOI:** 10.1155/bn/2951294

**Published:** 2025-02-25

**Authors:** David James, Vicky T. Lam, Booil Jo, Lawrence K. Fung

**Affiliations:** Department of Psychiatry and Behavioral Sciences, Stanford University, Palo Alto, California, USA

**Keywords:** autism, cortical thickness, postcentral gyrus, sensory

## Abstract

**Background:** Autism spectrum disorder (ASD) involves alterations in both cortical morphology and sensory processing. These structural and perceptual changes may lie on a continuum with typically developing (TD) individuals. However, investigations on possible links between these two factors are lacking, and it remains to be seen if their relationship differs by sex. We hypothesized that cortical thickness in the postcentral gyrus (a somatosensory processing hub) would correlate with sensory processing symptoms in a combined cohort of autistic and TD individuals. We also hypothesized that these correlations would differ based on sex.

**Methods:** We studied 23 autistic adults and 27 TD adults using magnetic resonance imaging to measure the cortical thickness of the postcentral gyrus and the Ritvo Autism Asperger Diagnostic Scale–Revised (RAADS-R) to measure autism characteristics, with a particular focus on the sensory motor subscale.

**Results:** The left postcentral gyrus (PCG) was found to be thicker in the autism group than in the TD group (*d* = 0.946, *p* = 0.003), particularly in autistic males compared to TD males and TD females. The RAADS-R sensory motor subscale and bilateral PCG cortical thickness were positively correlated across both autistic and TD males (Spearman's rho = 0.481, *p* = 0.008) but not females. These correlations were specific to the sensory motor subscale, as no correlations were found for RAADS-R total score or any of the other subscales.

**Conclusions:** These results demonstrate sex-specific differences in the relationship between cortical thickness at the PCG and sensory processing in autistic individuals and that these differences exist along a continuum that extends into the TD population. Our findings contribute to furthering our understanding of sex-specific neuroanatomical differences in people on the autism spectrum. The left PCG thickness could be a potential sex-specific biomarker for sensorimotor function that is generally applicable in both neurotypical and autism populations. With further validations, this biomarker could be used to track responses to interventions targeting sensorimotor challenges in people on the autism spectrum.

## 1. Introduction

Alterations in sensory processing represent a core diagnostic feature of autism spectrum disorder (ASD) [1]. Nearly 95% of adults on the autism spectrum report atypical levels of sensory processing [2], and a large percentage report “negative sensory responses” [3]. Sensory processing differences often correlate with increased social and communication differences [4], indicating a clinical need for research and intervention. The biological mechanisms underlying sensory differences in autism remain unclear. The association of cortical thickness to autism characteristics was investigated in several studies [5–8], but only a handful of studies have examined potential links between neuroanatomy and atypical sensory processing in autistic individuals [9, 10]. Furthermore, few studies have examined how this potential link may be influenced by biological sex.

Sex differences in the associations between cortical architecture and autism traits have been reported in the past decade. Atypical neuroanatomical features were found to have minimal spatial overlap in males and females [11]. Cortical neuroanatomy in the bilateral parahippocampal and entorhinal cortex, the fusiform and lingual gyrus, and the inferior or middle temporal lobe of autistic individuals were modulated by biological sex [12]. In a large-scale, multisite study, distinct patterns of neuroanatomic differences between males and females on the autism spectrum were found. While females had greater cortical thickness in the precentral gyrus and postcentral gyrus (PCG), males had greater cortical thickness in the right PCG [5]. These findings were consistent with our group's results, which showed the highest group (autistic adults vs. typically developing (TD) adults) differences in functional connectivity between PCG (both left and right) and the right thalamus [13]. Furthermore, we found that the functional connectivity of the PCG with the thalamus varies with the autism spectrum quotient (AQ) score in a sex-specific manner [13]. AQ was found to be positively correlated with the functional connectivity between the PCG and the right thalamus in autistic males but not in autistic females. Sensorimotor differences are also partially assessed in AQ.

The PCG is functionally the sensorimotor cortex (SM1). Given the converging evidence in the relevance of the PCG in adults on the autism spectrum, we conducted the current study with two main objectives. First, we aimed to replicate cortical thickness differences in PCG in a sample of adults on the autism spectrum. Second, given PCG's role in somatosensory processing, we sought to determine if cortical thickness was correlated with sensory symptoms, as measured by the Ritvo Autism Asperger Diagnostic Scale–Revised (RAADS-R) sensory motor subscale, in a sex-dependent manner.

This study is aimed at demonstrating the potential for the left PCG thickness to be a sex-specific biomarker for sensorimotor function in both neurotypical and autism populations. The findings of this study are significant as they will contribute to the advancement of personalized approaches in the field of autism. The PCG thickness could be used to as an objective neural measure of sensorimotor function differences in people on the spectrum. We could also use the left PCG thickness to track responses to interventions targeting sensorimotor challenges in people on the autism spectrum.

## 2. Methods

### 2.1. Participants

Twenty-eight autistic individuals (mean ± SD: 26.6 ± 8.3 years; 11 females; IQ 102.1 ± 16.5) and 29 sex- and IQ-matched TD individuals (27.7 ± 7.4 years; 10 females; IQ 112.1 ± 13.1) were recruited. One limitation of the study was that there was age difference between autistic males (23.6 ± 4.4 years) and autistic females (32.7 ± 9.7 years). Therefore, age could be a potential confounding factor in the data analyses. Due to poor image quality, five autistic participants and two TD participants were removed from the analysis. The Institutional Review Board of Stanford University reviewed and approved the methods of the present study. Written informed consent was provided by each participant. Inclusion criteria included the following: ASD diagnosis verified in a clinical interview based on DSM-5 criteria of ASD by a trained clinician, ASD category evaluated by the Autism Diagnostic Observation Schedule (ADOS) and Autism Diagnostic Inventory–Revised (ADI-R), 18 to 55 years of age, and full-scale IQ greater than or equal to 70. Potential participants were excluded if they were not physically healthy, had a history of chronic medical problems, or had a diagnosis of genetic syndrome. They would also be excluded if they were going through significant stressful situations. Additional exclusion criteria included preterm birth, low birth weight, severe mental health conditions such as schizophrenia, history of substance use disorder or alcohol use disorder, use of benzodiazepines during the study, blindness, deafness, pregnancy, and contraindication for neuroimaging studies. As this study was part of a larger investigation which included measuring GABA_A_ receptor density by positron emission tomography with [^18^F] flumazenil, we also excluded participants who took medications interacting with GABA metabolism and medications binding the GABA_A_ receptor. Further details on eligibility criteria were described in our previous publication [14, 15].

### 2.2. Behavioral Assessment

We used the sensory motor subscale of the RAADS-R [16] over other instruments because the sensory motor subscale of RAADS-R is more comprehensive in assessing sensory motor functioning (it has 20 items encompassing sensation avoiding, sensation seeking, sensory sensitive, and low registration in several sensory modalities) than other measures (ADOS, ADI-R, sensory responsiveness scale) that were included in our original study [14, 15]. The RAADS-R is a self-report questionnaire that assesses four symptom areas: language, social relatedness, circumscribed interests, and sensory motor. The Cronbach alpha coefficients for subscales of RAADS-R were satisfactory (social relatedness = 0.93, circumscribed interests = 0.95, sensory motor = 0.87, social anxiety = 0.89), indicating good internal consistency [16]. In a recent review of the literature field, RAADS-R was found to have satisfactory psychometric properties [17].

### 2.3. Neuroimaging Data Acquisition

Structural magnetic resonance (MR) images were acquired using a state-of-the-art simultaneous hybrid PET/MR imaging system (SIGNA PET/MR, GE Healthcare, Waukesha, Wisconsin). The following 3D T1-weighted protocol was used for high-resolution structural MR acquisition: repetition time (TR) = 7.9 ms; echo time (TE) = 2.9 ms; field of view (FOV) = 240 mm × 192 mm; matrix = 220 × 160; flip angle (FA) = 12°; axial plane; slice thickness (TH) = 1.4 mm; 128 slices.

### 2.4. MRI Data Analysis

Cortical thickness from T1-weighted MR images was determined by FreeSurfer (Version 6.0.0; [18]). The *recon-all* function within FreeSurfer was used to process all T1-weighted images. The pipeline included skull stripping, segmentation of gray matter from white matter, normalization of signal intensity, registration of surface atlas, and surface extraction. To improve the quality of the surface extraction, the locations of the pial surfaces and/or gray matter/white matter surfaces were manually adjusted. The first author was the only rater, so there was no need to determine interrater reliability Among the images of 28 autistic and 29 TD participants, those corresponding to five autistic and two TD male participants were removed due to suboptimal image quality. Fifty individuals remained for further data analysis. Cortical thickness values defined by the Hammersmith atlas were extracted by the mri_vol2surf function in FreeSurfer.

### 2.5. Statistical Analysis

ROI analyses were run in SPSS 25. *t*-tests were used to assess for differences in cortical thickness between autistic and TD groups. One-way ANOVA was used to assess differences in cortical thickness across four sex + diagnosis groups (autistic males, autistic females, TD males, TD females). Post hoc Tukey HSD was performed on ANOVA results. Correlations between RAADS-R measures and cortical thickness were assessed using Spearman's rho. Participants were separated by sex, and within each sex, autistic and TD participants were combined together to calculate rho values. To verify the statistical assumptions of Spearman's rho, we ensured that the data involved continuous variables, paired observations, and (by visual inspection of the scatterplot) had a monotonic relationship. To verify assumptions of one-way ANOVA, we used the “explore” function in SPSS to assess for the presence of outliers, normally distributed dependent variables for each independent variable group, and homogeneity of variances. We have two ROIs (left PCG and right PCG). To correct for multiple comparisons, we have set significance for *t*-tests, one-way ANOVA, and Spearman's rho at *p* < 0.025, after Bonferroni correction.

### 2.6. Power Analysis

According to a report by Scheel et al., the mean cortical thickness of a cluster containing the right precentral gyrus, PCG, and supramarginal gyrus is 2.03 ± 0.12(SD) in high-functioning adults with autism and 2.12 ± 0.17 in TD [19]. Using this information, we needed a sample size of 21 individuals in each group to achieve a power of 80% (*α* = 0.05) in a *t*-test comparing two independent means. As part of the procedure to verify assumptions for the one-way ANOVA, we assessed for outliers in the sex-stratified analyses. Outliers were found for TD females (one outlier) and TD males (one outlier) at the left postcentral gyrus (Figure S1) and for ASD males (one outlier) at the right postcentral gyrus (Figure S2).

## 3. Results

Demographic information for study participants is shown in Table 1. Group differences were observed in full-scale IQ (*F*(1, 46) = 5.009, *p* = 0.030) but not sex (*X*^2^(1, *N* = 50) = 0.593, *p* = 0.441) or age (*F*(1, 48) = 0.187, *p* = 0.667). As expected, significant differences were seen between autistic and TD groups for RAADS-R total score and on the sensory motor, social relatedness, circumscribed interests, and language subscales. These differences persisted when the data was stratified into sex + diagnosis groups.

The mean thickness of the left PCG was greater in the autism group compared to the TD group; the mean thickness of the right PCG was also larger in the autism group (Table 2). Only the difference at the left PCG was still significant after Bonferroni correction for two ROIs (*p* < 0.025). The cortical thickness of the left PCG differed across sex and diagnosis groups (Table 3). Post hoc analysis at the left PCG revealed significant differences between autistic males and TD males and between autistic males and TD females. No significant differences were found at the right PCG (Table 4).

**Figure 1:**
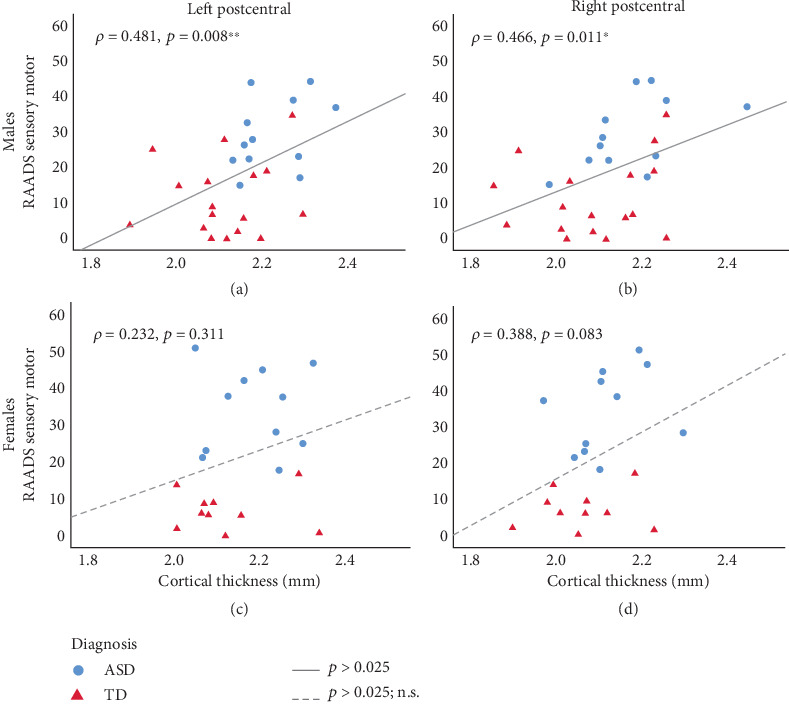
Sex differences in the relationship between sensory motor symptoms and cortical thickness at the postcentral gyrus. When both autistic and TD males are combined together, positive correlations (Spearman's rho) are seen between cortical thickness and RAADS sensory motor subscale at the left (a) and right (b) postcentral gyrus. These correlations are still significant after Bonferroni correction for two ROIs with alpha threshold of 0.025 (solid lines indicate significant correlations). Positive correlations are seen for females (c and d), but these are not statistically significant (dashed lines indicate nonsignificant correlations).

For males, a positive correlation between cortical thickness and RAADS-R sensory motor subscale was seen at both the left PCG and right PCG in both autistic and TD groups (Figures 1(a) and 1(b)). Thicker PCG was associated with more sensory motor differences in males. Interestingly, this correlation was specific to the sensory motor subscale, as neither the RAADS-R total score nor any of the other subscales (social relatedness, circumscribed interests, or language) were significantly correlated with cortical thickness (Table 5). For females, whether autism or TD, no significant correlations were seen between cortical thickness and any of the RAADS-R subscales or total score (Figures 1(c) and 1(d) and Table 5).

## 4. Discussion

The aim of this study was to assess the relationship between PCG cortical thickness and sensory motor symptoms in autistic individuals. Cortical thickness at the left PCG was found to be different between autistic and TD groups, with most of this difference due to differences between autistic and TD males and between autistic males and TD females. When autistic and TD groups were combined together, cortical thickness at bilateral PCG significantly correlated with RAADS-R sensory motor subscale scores in males but not females. Our findings support the idea that autism traits, at least for sensory processing, exist along a continuum that includes TD individuals. Furthermore, we propose that this continuum may differ based on sex.

Brain overgrowth in autism is one of the most consistent findings in neuroimaging studies of children on the autism spectrum [20]. Similarly, higher cortical thickness in autistic individuals has been reported [6]. Increased cortical thickness in autistic people may be explained by the increase in neuron size [21] and the decrease synaptic pruning associated with autism [22],

Previous studies have found correlations between neuroanatomy and autistic traits with conflicting results. In a longitudinal MRI study looking at cortical thickness changes across the lifespan, cortical thickness in several brain regions positively correlated with autism characteristics [6]. These significant correlations were seen in the primary somatosensory and motor cortices, including the left PCG and precentral gyrus. In contrast, in another study, negative correlations were found between autism traits and cortical thickness at the medial orbitofrontal gyrus, PCG, and lingual gyrus [9]. When comparing the positive correlations in the present study with conflicting prior literature, we should remember that each of these studies uses different measures of autism traits. Khundrakpam et al. measured total autism characteristics by ADOS; Gebauer et al. assessed overall autism traits by AQ. In the present study, we use the sensory motor subscale of RAADS-R, as we focus on the sensory motor phenotype.

The current study also adds to the literature on autism characteristics in the TD population, supporting the idea that autism traits exist on a continuum that extends into the TD population [23]. In Gebauer et al.'s study mentioned above, significant correlations were seen when autistic and TD groups were combined together [9], as done in the present study. Together, these findings support the idea of a broader autism phenotype (BAP) proposed by several authors [24, 25]. Studying this phenotype in TD individuals and/or pooling TD individuals into a common analysis cohort with autistic individuals may provide insight into the origins of specific autism characteristics.

Our finding that cortical thickness and sensory motor symptoms correlate in males but not females is consistent with prior literature on sex differences in autism. In previous studies on adults, sex differences have been observed in the RAADS-R sensory motor domain, with autistic females scoring higher on this subscale than autistic males [26, 27]. Our group's recent study on thalamocortical connectivity, discussed above, found that connectivity between the right thalamus and left pre/postcentral gyrus positively correlates with AQ total score in male adults on the spectrum [13]. In autistic females, a negative correlation was seen, but this was not statistically significant, given the limited sample size. In the current study, Figure 1 shows virtually no overlap between autistic and TD groups in females (blue vs. red data points), whereas there is more of a gradual overlap between these groups in males, suggesting that the continuous correlation between cortical thickness and sensorimotor symptoms between autistic and TD individuals may hold true for males but not females. Sex-specific differences in cortical development in autism could explain this finding. A recent study by Andrews et al. found that there are more prominent differences in cortical thickness and thinning over the lifespan between autistic and neurotypical females than between autistic and neurotypical males [28]. This is in line with the liability threshold model of autism that hypothesizes that females require a greater number of risk factors to manifest autism than males [29]. This could explain why we see two separate groups in our female sample in Figure 1 that are not correlated in the same linear fashion as the male sample. Additionally, the impact of the menstrual cycle is another confounder that could make it difficult to detect potential correlations between cortical thickness and sensorimotor symptoms in females. Meeker et al. found that the somatosensory cortex is thinner during the follicular compared to the menstrual phase, a time when women showed greater sensory sensitivity to electrical stimuli [30]. Future studies may need to control for the menstrual cycle phase to better detect potential correlations between somatosensory cortex and sensory sensitivity in females with autism. Taken together, our current findings suggest that the mechanisms underlying atypical sensory processing in autistic individuals are sex dependent. However, caution should be used when comparing these studies. Many of these studies use different questionnaires to characterize autism characteristics, and not all questionnaires emphasize sensory processing to the same degree. For instance, the AQ does not have a sensory motor subscale like the RAADS-R.

Our study has several limitations. Our small sample size, particularly for the sex comparisons, might contribute to Type II errors. This issue could explain why we do not see significant correlations between cortical thickness and sensory motor symptoms in the female cohort. Future studies to replicate these results should increase sample size accordingly. We also have fewer female participants than male participants, so future studies should also recruit more balanced cohorts with respect to sex. To address whether sample size imbalances between male and female participants could have impacted the power for sex-stratified analyses, we looked for outliers in the cortical thickness data and conducted one-way ANOVAs both with and without the outliers (Tables S1 and S2). The presence or absence of outliers did not change the results. Furthermore, the age between autistic males and autistic females was not well matched, with an average difference of 9 years between the groups. As cortical thickness varies with age [5], future studies should ensure improved age matching so that the same phases of cortical aging are compared between groups. IQ was also significantly different between autistic and TD adults. While it is not ideal to have significantly different mean IQs between groups, when we reran our analyses looking at the relationship between sex × diagnosis group and cortical thickness using a general linear model with IQ and age as covariates, we still saw statistically significant differences from the sex + diagnosis factor (Tables S3 and S4 for the left PCG and Tables S5 and S6 for the right PCG). Finally, while the current study is focused solely on structural imaging, prior studies have shown that there are sex differences in functional connectivity in autism [31, 32]. It will be important for future studies to integrate structural imaging with task-based functional analyses to obtain a more comprehensive understanding of the sex differences in autism. For example, future studies could look for sex differences in postcentral gyrus activation during tasks that involve discrimination of somatosensory stimuli; similar studies have already been done but without assessing for sex differences [33].

To the best of our knowledge, this is the first study to examine sex differences in the relationship between cortical morphology of the somatosensory cortex and sensory motor symptoms in a combined cohort of autistic and TD adults. The left PCG was found to be thicker in autistic individuals, a difference that was primarily driven by autistic males. Furthermore, we found significant correlations between cortical thickness of the left PCG and sensory motor symptoms in males but not females when autism and TD groups were combined together. Overall, these results show evidence for sex-specific differences in a neuroanatomical correlate of atypical sensory processing in autistic individuals and that these differences exist along a continuum that extends into the TD population. Our findings suggest that the left PCG thickness could be a potential sex-specific biomarker for sensorimotor function that is generally applicable in both neurotypical and autism populations. This biomarker has the potential to be used in tracking responses to interventions targeting sensorimotor challenges in people on the autism spectrum. Replication of these results is warranted.

## Figures and Tables

**Table 1:** Participant characteristics.

	**Autism male and female ** **(** **N** = 23**)**	**TD male and female ** **(** **N** = 27**)**	**Autism male ** **(** **N** = 12**)**	**Autism female ** **(** **N** = 11**)**	**TD male ** **(** **N** = 17**)**	**TD female ** **(** **N** = 10**)**	**Autism vs. TD**		**Sex ± diagnosis**	
							**t** ** or ** **X** ^2^	**p**	**ANOVA ** **F**	**p**
Age (years)	28.0 ± 8.6	27.0 ± 7.0	23.6 ± 4.4	32.7 ± 9.7	26.1 ± 6.5	28.6 ± 7.9	0.433	0.667	3.406	0.025
Male (%)	52.2	63	100	0	100	0	0.593	0.441	n/a	n/a
FSIQ	103.4 ± 25.3	118.3 ± 20.1	104.4 ± 27.5	102.3 ± 24.1	122.0 ± 19.6	113.1 ± 20.6	−2.238	0.030	1.935	0.138
RAADS										
Total	127.8 ± 35.8	47.2 ± 36.0	114.6 ± 35.8	142.3 ± 31.1	55.3 ± 41.9	33.5 ± 17.2	7.911	< 0.001	24.781	< 0.001
Sensory motor	31.5 ± 10.8	9.8 ± 9.2	29.2 ± 10.1	34.1 ± 11.5	11.4 ± 10.7	7.0 ± 5.5	7.658	< 0.001	20.695	< 0.001
Social relatedness	61.9 ± 20.2	23.3 ± 19.3	55.5 ± 22.5	68.8 ± 15.4	28.1 ± 22.6	15.2 ± 7.4	6.888	< 0.001	18.954	< 0.001
Circumscribed interests	23.8 ± 10.1	9.4 ± 6.8	19.9 ± 7.6	28.0 ± 11.1	11.1 ± 7.3	6.6 ± 5.1	5.98	< 0.001	15.971	< 0.001
Language	10.3 ± 4.4	4.4 ± 4.0	9.6 ± 4.8	11.1 ± 4.0	4.9 ± 4.5	3.7 ± 2.9	4.938	< 0.001	8.407	< 0.001

*Note:* Reported values are mean ± SD. For all autistic versus all TD comparisons, Student's *t*-test was used for continuous variables and chi-square for categorical variables.

**Table 2:** Cortical thickness comparisons at the postcentral gyrus stratified by diagnosis.

**Hemisphere**	**All autism (** **N** = 23**)**	**All TD (** **N** = 27**)**	**t**	**p**
Left	2.20 ± 0.09	2.11 ± 0.10	3.137	0.003⁣^∗∗^
Right	2.14 ± 0.11	2.08 ± 0.12	2.153	0.036

*Note:* Reported values are mean ± SD.

⁣^∗∗^*p* < 0.005.

**Table 3:** Cortical thickness comparisons at the postcentral gyrus stratified by sex and diagnosis.

**Hemisphere**	**Autism male (** **N** = 12**)**	**Autism female (** **N** = 11**)**	**TD male (** **N** = 17**)**	**TD female (** **N** = 10**)**	**ANOVA ** **F**	**p**
Left	2.22 ± 0.08	2.17 ± 0.09	2.11 ± 0.11	2.11 ± 0.11	3.785	0.017⁣^∗^
Right	2.17 ± 0.12	2.12 ± 0.09	2.09 ± 0.13	2.06 ± 0.10	2.158	0.106

*Note:* Reported values are mean ± SD.

⁣^∗^*p* < 0.05.

**Table 4:** Sex + diagnosis cortical thickness differences using post hoc Tukey HSD.

	**Autism male**	**TD male**	**Autism female**	**TD female**
Left postcentral gyrus				
Autism male	0	−0.109⁣^∗^	−0.051	−0.113⁣^∗^
TD male		0	0.058	−0.003
Autism female			0	−0.062
TD female				0
Right postcentral gyrus				
Autism male	0	−0.083	−0.055	−0.114
TD male		0	0.028	−0.030
Autism female			0	−0.058
TD female				0

*Note:* Mean differences shown (columns–rows).

⁣^∗^*p* < 0.05.

**Table 5:** Correlation coefficients between cortical thickness of postcentral gyri and RAADS subscales.

**Region of interest**	**Sex**	**Social relatedness**	**Circumscribed interests**	**Language**	**Total score**
Left postcentral	M	0.256	0.234	0.116	0.313
Right postcentral	M	0.197	0.138	0.13	0.259
Left postcentral	F	0.303	0.161	0.152	0.19
Right postcentral	F	0.415	0.383	0.352	0.43

*Note:* Shown values represent Spearman's rho correlations between cortical thickness at a given region of interest (leftmost column) and performance on a RAADS subscale (Columns 3–5) or RAADS total score (Column 6). Autistic and TD cohorts are combined for each correlation. Correlation coefficients between cortical thickness at a given region of interest and RAADS sensory motor subscale are reported in Figure 1.

## Data Availability

The data presented in this manuscript will be available upon request.

## Supporting Information

Supporting Information Additional supporting information can be found online in the Supporting Information section. Figure S1: Cortical thickness at the left postcentral gyrus (LPCG) by sex + diagnosis group. Outliers are identified by case number. Table S1: One-way ANOVAs comparing cortical thickness across sex + diagnosis groups at LPCG. Figure S2: Cortical thickness at the right postcentral gyrus (RPCG) by sex + diagnosis group. Outliers are identified by case number. Table S2: One-way ANOVAs comparing cortical thickness across sex + diagnosis groups at RPCG. Table S3: General linear model of the relationship between cortical thickness and sex + diagnosis group with IQ and age as covariates (left postcentral gyrus). Table S4: Pairwise cortical thickness comparisons among sex + diagnosis groups at the left postcentral gyrus. Table S5: General linear model of relationship between cortical thickness and sex + diagnosis group with IQ and age as covariates (right postcentral gyrus). Table S6: Pairwise cortical thickness comparisons among sex + diagnosis groups at the right postcentral gyrus.
