# Supplementary material for: Sex Differences in the Relationship Between Cortical Thickness and Sensory Motor Symptoms in Adults on the Autism Spectrum
Source: Behav Neurol. 2025 Feb 25;2025:2951294. doi: 10.1155/bn/2951294 (PMC11879536; doi:10.1155/bn/2951294)
Supplement: Supporting Information — Additional supporting information can be found online in the Supporting Information section. Figure S1: Cortical thickness at the left postcentral gyrus (LPCG) by sex + diagnosis group. Outliers are identified by case number. Table S1: One-way ANOVAs comparing cortical thickness across sex + diagnosis groups at LPCG. Figure S2: Cortical thickness at the right postcentral gyrus (RPCG) by sex + diagnosis group. Outliers are identified by case number. Table S2: One-way ANOVAs comparing cortical thickness across sex + diagnosis groups at RPCG. Table S3: General linear model of the relationship between cortical thickness and sex + diagnosis group with IQ and age as covariates (left postcentral gyrus). Table S4: Pairwise cortical thickness comparisons among sex + diagnosis groups at the left postcentral gyrus. Table S5: General linear model of relationship between cortical thickness and sex + diagnosis group with IQ and age as covariates (right postcentral gyrus). Table S6: Pairwise cortical thickness comparisons among sex + diagnosis groups at the right postcentral gyrus. [file 2951294.f1.docx]

**Supplemental Figure 1 –** Cortical thickness at left postcentral gyrus (LPCG) by sex + diagnosis group. Outliers are identified by case number.

**
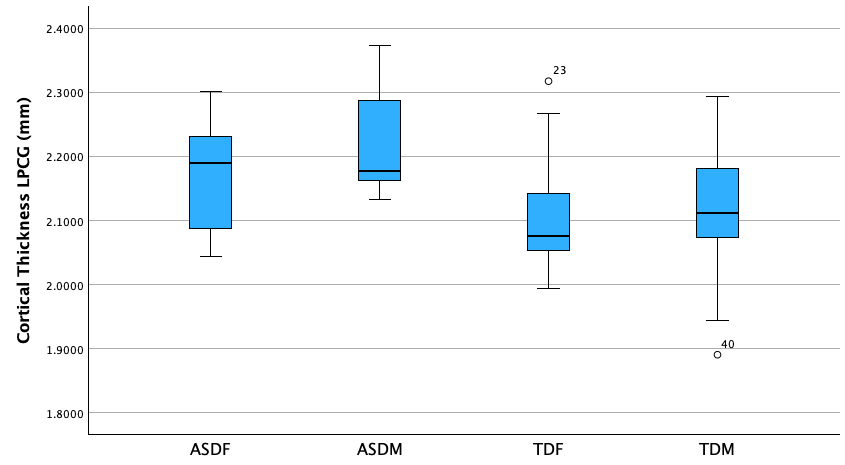
**

**Supplemental Table 1 –** One-way ANOVAs comparing cortical thickness across sex + diagnosis groups at LPCG.

*With outliers*

**
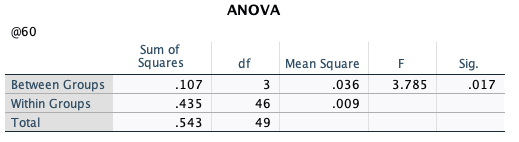
**

*Without outliers*

**
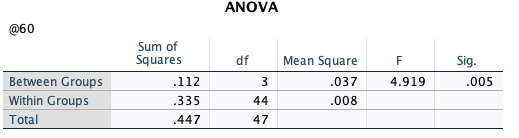
**

**Supplemental Figure 2 –** Cortical thickness at right postcentral gyrus (RPCG) by sex + diagnosis group. Outliers are identified by case number.

**
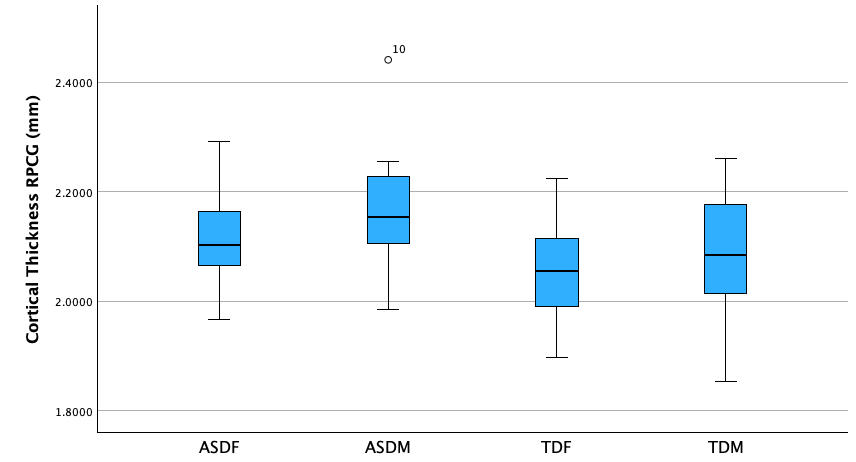
**

**Supplemental Table 2 -** One-way ANOVAs comparing cortical thickness across sex + diagnosis groups at RPCG.

*With outliers*

**
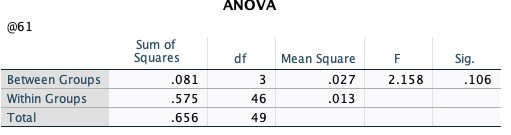
**

*Without outliers*

**
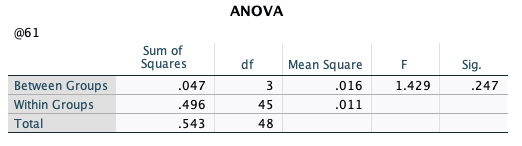
**

**Supplemental Table 3 –** General linear model of the relationship between cortical thickness and sex + diagnosis group with IQ and age as covariates (Left Postcentral Gyrus).


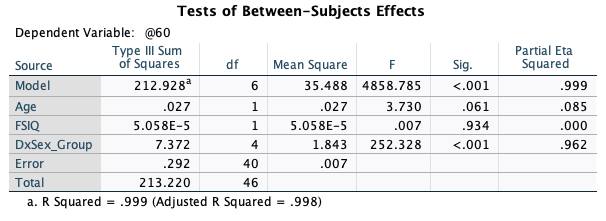


**Supplemental Table 4 –** Pairwise cortical thickness comparisons amongst sex + diagnosis groups at the Left Postcentral Gyrus


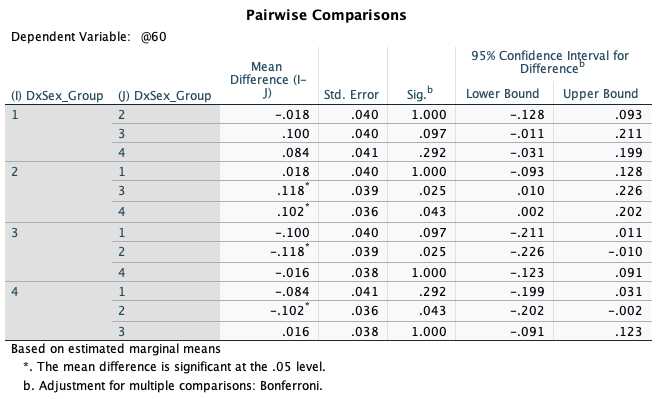


Legend:

1 = ASD Female

2 = ASD Male

3 = TD Female

4 = TD Male

**Supplemental Table 5** – General linear model of relationship between cortical thickness and sex + diagnosis group with IQ and age as covariates (Right Postcentral Gyrus)


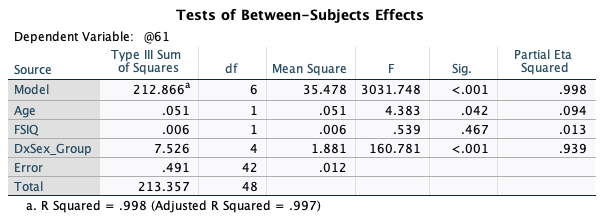


**Supplemental Table 6 –** Pairwise cortical thickness comparisons amongst sex + diagnosis groups at the Right Postcentral Gyrus.


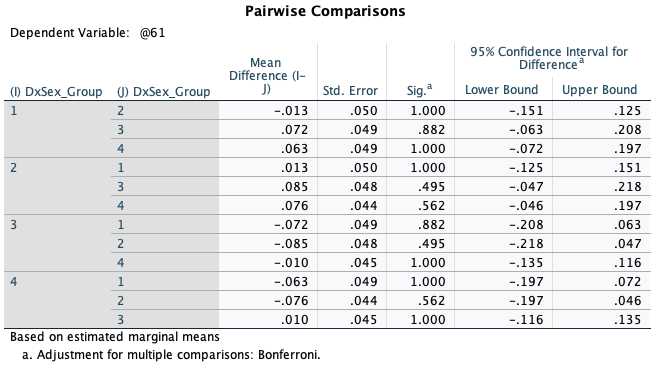


Legend:

1 = ASD Female

2 = ASD Male

3 = TD Female

4 = TD Male
